# Pseudomyxoma Peritonei: A Case Report

**DOI:** 10.7759/cureus.113307

**Published:** 2026-07-24

**Authors:** Helena Awada, Christian Dean, Catherine Kim, Sylvia Li, Sarkis Arabian

**Affiliations:** 1 Internal Medicine, Arrowhead Regional Medical Center, Colton, USA; 2 Emergency Medicine, Arrowhead Regional Medical Center, Colton, USA; 3 Medicine, California University of Science and Medicine, Colton, USA; 4 Critical Care, Arrowhead Regional Medical Center, Colton, USA

**Keywords:** appendiceal mucinous neoplasm, cytoreductive surgery, hyperthermic intraperitoneal chemotherapy, mucinous ascites, peritoneal carcinomatosis, pseudomyxoma peritonei, pulmonology and critical care

## Abstract

Pseudomyxoma peritonei (PMP) is a rare clinicopathologic syndrome characterized by progressive accumulation of mucinous ascites within the peritoneal cavity, most commonly arising from an appendiceal mucinous neoplasm. Diagnosis is frequently delayed because of nonspecific symptoms and variable radiographic findings. We present a case of high-grade PMP in a 41-year-old man whose initial presentation was notable for atypical endocrine and genitourinary symptoms, including progressive gynecomastia, ejaculatory dysfunction, pelvic pain, and hydrocele formation. Initial abdominal magnetic resonance imaging (MRI) was unremarkable; however, subsequent computed tomography demonstrated loculated ascites and a large abdominal mass. Exploratory laparotomy revealed extensive peritoneal disease with mucinous adenocarcinoma and large-volume malignant ascites. The patient subsequently developed radiographic findings concerning for thoracic involvement, including bilateral loculated pleural effusions and mediastinal abnormalities, with respiratory failure requiring prolonged mechanical ventilation. The diagnosis of PMP had previously been established during exploratory laparotomy at an outside institution, where malignant ascitic-fluid cytology and an intraoperative biopsy demonstrated mucinous adenocarcinoma. Because the complete outside pathology report was unavailable following transfer, additional tissue sampling was pursued. Thoracentesis, abdominal fluid drainage, and endobronchial ultrasound-guided sampling were nondiagnostic, whereas diagnostic laparoscopy subsequently provided confirmatory tissue demonstrating high-grade mucinous adenocarcinoma with positive CK7, CK20, CDX2, and SATB2 immunostaining, supporting lower gastrointestinal differentiation. However, a definitive primary tumor site could not be established based on the available clinical, operative, and pathologic information. Owing to unresectable peritoneal disease, ventilator-dependent respiratory failure, severe chronic illness-related malnutrition, declining functional status, and radiographic findings concerning for extra-abdominal involvement, the patient was not a candidate for cytoreductive surgery combined with hyperthermic intraperitoneal chemotherapy. Following multidisciplinary discussion, he received one cycle of palliative FOLFOXIRI (5-fluorouracil, leucovorin, oxaliplatin, and irinotecan). No further chemotherapy was administered because of persistent ventilator dependence, *Enterobacterales* and *Serratia* bacteremia requiring vasopressor support, and continued clinical deterioration, and the patient died shortly thereafter. This case highlights several uncommon features of PMP, including atypical initial manifestations, initially negative imaging, radiographic findings concerning for thoracic involvement, and repeated false-negative cytologic studies. It underscores the importance of obtaining adequate tissue for histopathologic confirmation when prior pathology is unavailable or minimally invasive studies are nondiagnostic, as well as early referral to specialized centers before disease progression precludes potentially curative treatment.

## Introduction

Pseudomyxoma peritonei (PMP) is a rare malignant condition characterized by dissemination of mucin-producing neoplastic cells throughout the peritoneal cavity, resulting in progressive accumulation of gelatinous ascites [1,2]. The disease most commonly originates from a perforated appendiceal mucinous neoplasm, although ovarian, colorectal, pancreatic, and other gastrointestinal primary sites have been reported [1].

Management of PMP has evolved substantially over the past several decades. Cytoreductive surgery combined with hyperthermic intraperitoneal chemotherapy (CRS-HIPEC) has become the standard treatment approach for appropriately selected patients and has significantly improved long-term outcomes compared with historical debulking procedures [3].

PMP was originally described as a distinct clinicopathologic syndrome associated with progressive intraperitoneal accumulation of mucinous tumor deposits [4]. The estimated incidence is approximately two to four cases per million individuals annually, making PMP an uncommon but clinically significant entity [5]. Patients frequently present with vague and nonspecific symptoms, including abdominal distension, bloating, abdominal pain, early satiety, and weight loss [6]. Although abdominal symptoms predominate, unusual clinical presentations have been reported, including presentation as a scrotal mass, inguinal hernia, uterine prolapse, and findings initially attributed to gynecologic pathology [7-10]. Because the clinical manifestations of PMP overlap with numerous benign and malignant gastrointestinal disorders, diagnosis is often delayed until imaging studies or surgical exploration reveal characteristic mucinous ascites.

The pathogenesis of PMP remains incompletely understood. Current evidence suggests that disease behavior is primarily determined by the underlying mucinous neoplasm and its histopathologic characteristics. Microbial influences have also been investigated as potential modifiers of the tumor microenvironment. Although *Helicobacter pylori* is a well-established cause of gastric malignancies [11], no causal relationship with PMP has been established. Studies have identified distinct microbial populations within PMP that may influence tumor biology through mechanisms such as modulation of β-catenin signaling pathways [12,13].

Histologically, PMP encompasses a spectrum ranging from low-grade mucinous carcinoma peritonei, which generally follows a more indolent clinical course, to high-grade disease and signet-ring variants associated with increased recurrence rates and reduced survival [14-16]. Immunohistochemical markers including CDX2 and SATB2 are frequently used to support an appendiceal or colorectal origin and assist in diagnostic classification [17]. Thoracic involvement is uncommon but has been reported in advanced disease and may complicate management and prognosis [18].

Computed tomography (CT) remains the preferred imaging modality for evaluating disease extent and identifying features such as peritoneal implants, scalloping of visceral surfaces, and omental caking [19]. Disease recurrence remains common, and outcomes are strongly influenced by histologic grade, disease burden, and completeness of cytoreduction [5,15,16].

Given the rarity and heterogeneity of PMP, individual case reports continue to provide valuable insight into atypical clinical presentations, diagnostic challenges, and treatment limitations. We present a case of high-grade PMP notable for atypical endocrine and genitourinary symptoms that preceded recognition of the underlying malignancy, initially nondiagnostic MRI, repeated nondiagnostic cytologic studies despite an established diagnosis, radiographic findings concerning for thoracic involvement, and rapid clinical deterioration that ultimately precluded CRS-HIPEC.

## Case presentation

A 41-year-old man with a medical history significant for *Helicobacter pylori* gastritis and hypertension initially presented in 2023 with progressive gynecomastia, ejaculatory dysfunction, and pelvic pain. These symptoms preceded the diagnosis of PMP and prompted endocrine and genitourinary evaluation. Initial evaluation included breast ultrasonography demonstrating possible breast masses, scrotal ultrasonography revealing bilateral epidermal cysts and a complex right hydrocele, and MRI of the abdomen and pelvis performed in January 2024, which was reported as unremarkable. The original MRI images were not available for review upon transfer; therefore, the reported findings could not be independently verified, and differences in imaging technique or interpretation cannot be excluded.

**Figure 1:**
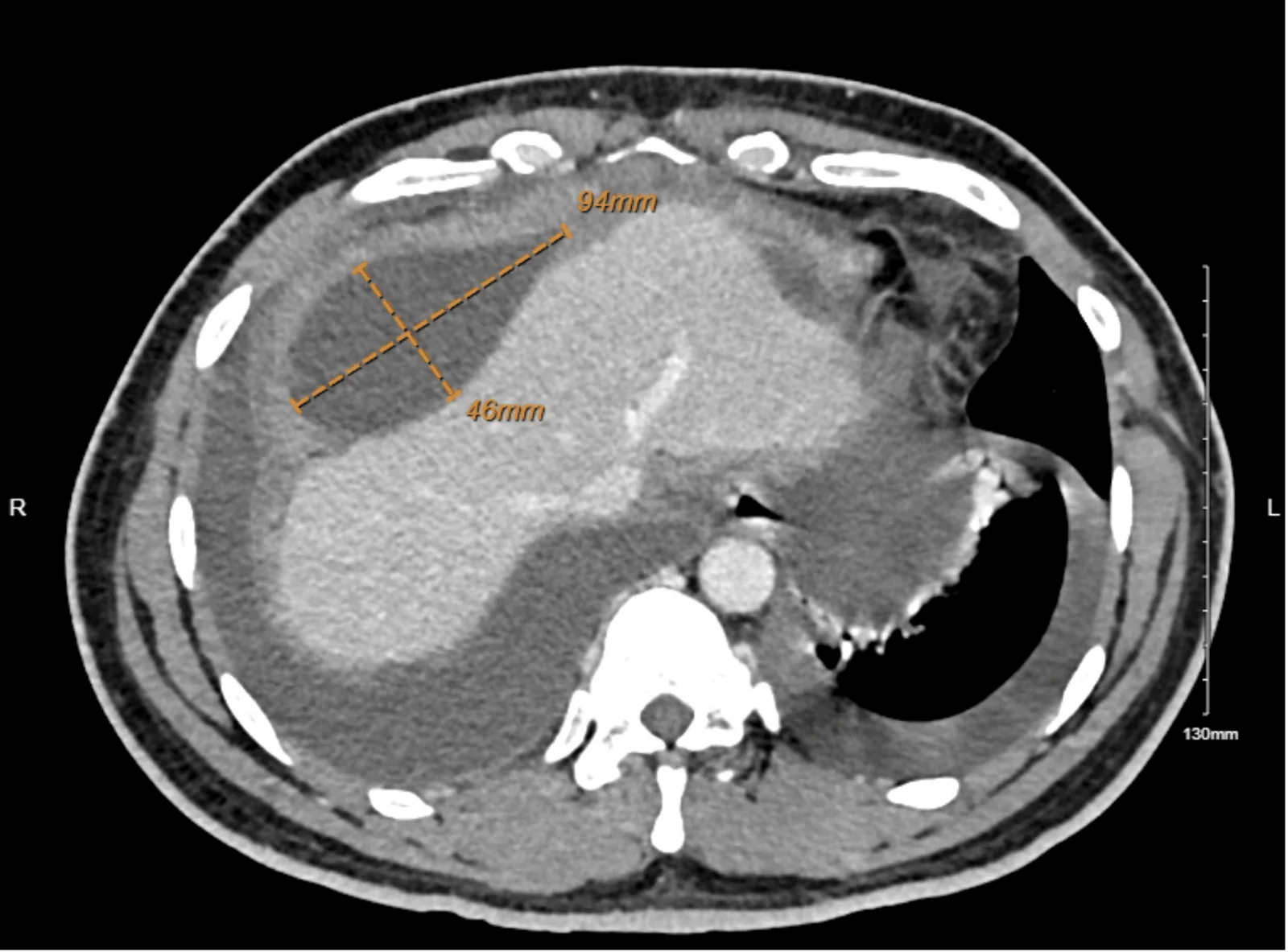
Contrast-Enhanced CT Abdomen and Pelvis Demonstrating Loculated Ascites Contrast-enhanced computed tomography (CT) of the abdomen and pelvis obtained in April 2024 demonstrating loculated ascites throughout the abdomen, including a large perihepatic fluid collection measuring 4.6 × 9.4 × 9.3 cm.

As the patient's symptoms progressed, contrast-enhanced CT of the abdomen and pelvis obtained in April 2024 demonstrated loculated ascites adjacent to the liver measuring 4.6 × 9.4 × 9.3 cm (Figure 1) and a soft tissue mass along the greater curvature of the stomach measuring 10.2 × 11.3 × 5.8 cm. By June 2024, he developed progressive abdominal distension secondary to ascites accompanied by unintentional weight loss.

According to records obtained from the University Hospital of the West Indies, the patient underwent exploratory laparotomy in February 2025, during which the diagnosis of PMP was established. Intraoperative findings included extensive omental caking with tumor deposits involving the small bowel, peritoneum, and liver. Approximately 11 L of ascitic fluid were evacuated. Cytologic analysis of the ascitic fluid was positive for malignant cells, and an intraoperative biopsy demonstrated mucinous adenocarcinoma. However, the complete pathology report and original histologic slides from the outside institution were unavailable for review at the receiving institution.

**Figure 2:**
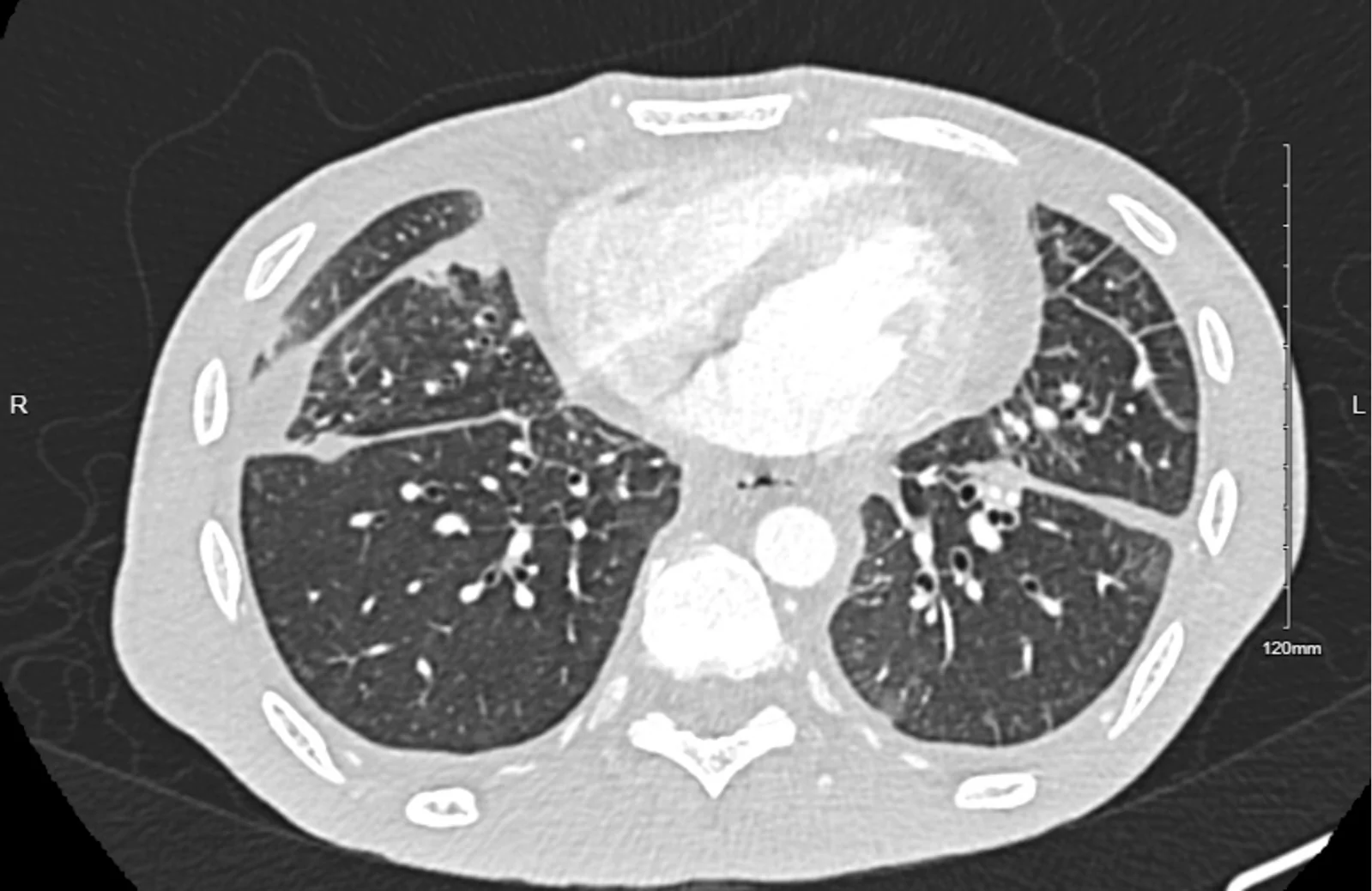
CT Angiogram of the Chest Demonstrating Right Middle Lobe Collapse and Bilateral Pleural Effusions Computed tomography angiography (CTA) of the chest obtained three days before hospital day 1, demonstrating collapse and consolidation of the medial segment of the right middle lobe with small bilateral pleural effusions.

Approximately five months after the initial diagnosis, the patient developed progressive respiratory symptoms. Three days before hospital day 1, CT angiography (CTA) of the chest demonstrated consolidation and collapse of the medial segment of the right middle lobe with small bilateral pleural effusions (Figure 2). Concurrent CT pulmonary angiography revealed mediastinal and hilar lymphadenopathy, bilateral loculated pleural effusions, and nodular soft tissue densities within the mediastinum and bilateral hilar. These imaging findings raised concern for thoracic extension of disease; however, subsequent pleural fluid cytology was negative for malignant cells, endobronchial ultrasound (EBUS) did not identify a suitable biopsy target, and pleural biopsy was not performed. Therefore, histologic confirmation of thoracic involvement was not obtained.

On hospital day 1, the patient re-presented to the University Hospital of the West Indies with worsening shortness of breath and a witnessed syncopal episode lasting approximately five minutes. He required emergent endotracheal intubation for airway protection in the setting of acute hypercapnic respiratory failure. At presentation, his Glasgow Coma Scale score was 3. Arterial blood gas analysis revealed a pH of 7.12, partial pressure of carbon dioxide (pCO₂) of 112.7 mmHg, and lactate level of 1.4 mmol/L. Respiratory cultures obtained on hospital day 2 grew *Enterobacter bugandensis*, which demonstrated susceptibility to gentamicin, piperacillin-tazobactam, and trimethoprim-sulfamethoxazole, and resistance to amoxicillin-clavulanate, ampicillin, and cefuroxime. The patient was treated with ceftriaxone and azithromycin.

Repeat CT imaging of the abdomen and pelvis obtained on hospital day 2 demonstrated persistent loculated ascites surrounding the liver measuring 5.4 × 9.3 × 10.8 cm, similar in appearance to prior imaging from April 2024; these imaging studies were not available upon transfer. Because of progressive disease and consideration for CRS-HIPEC, the patient was transferred to Arrowhead Regional Medical Center (ARMC) on hospital day 11 for further evaluation and management.

**Figure 3:**
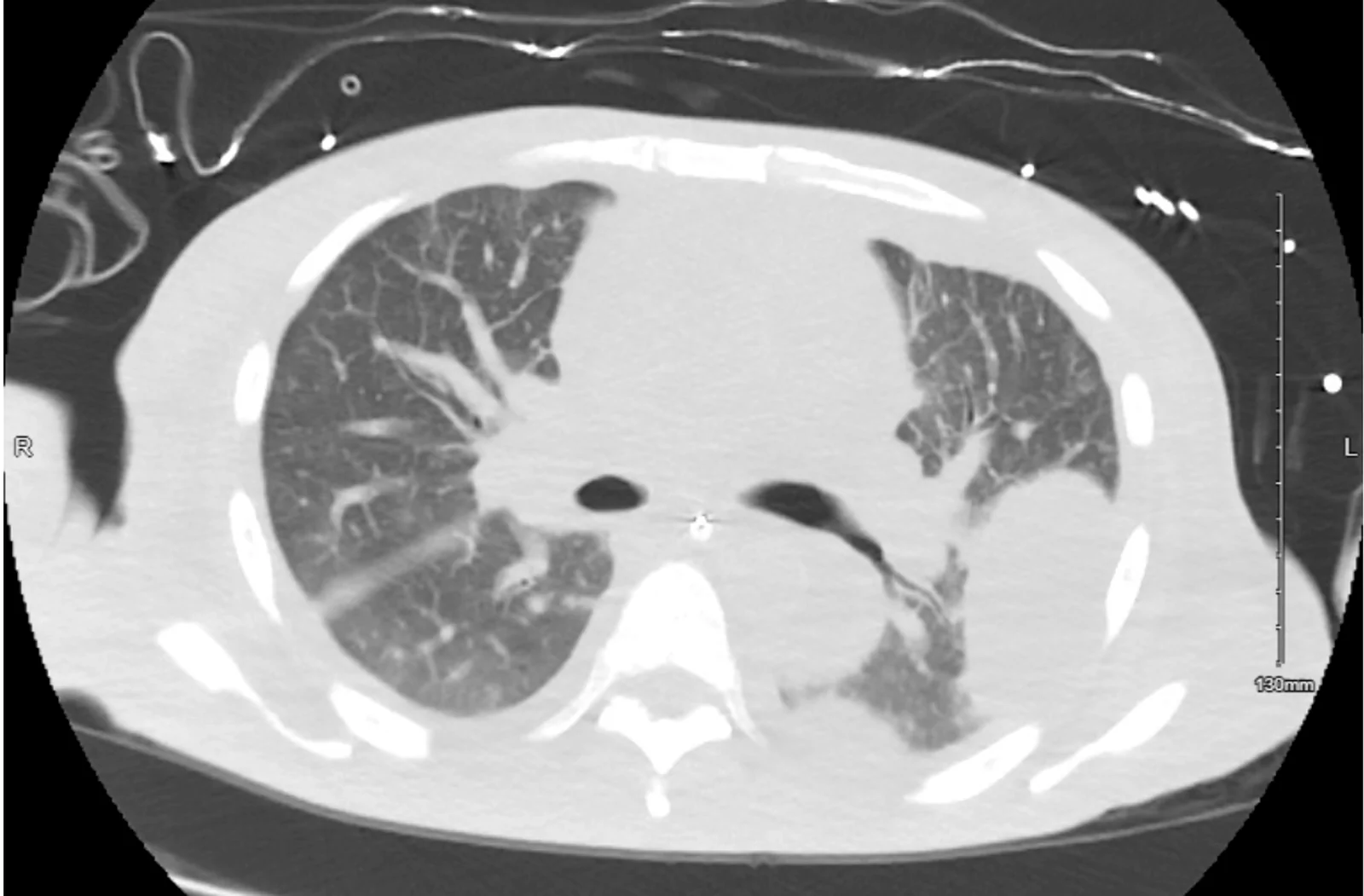
Non-contrast CT Chest and Abdomen Demonstrating Bilateral Loculated Pleural Effusions Computed tomography imaging demonstrating worsening bilateral loculated pleural effusions with largest component in the left pleural space, in the setting of advanced pseudomyxoma peritonei.

Upon arrival at ARMC, the patient was intubated, cachectic, and critically ill. Physical examination revealed a markedly distended abdomen, bilateral loculated pleural effusions, loculated ascites, and 1+ bilateral lower-extremity pitting edema. Laboratory evaluation demonstrated leukocytosis of 23.7 × 10⁹/L ( Figure 3). The patient was unable to tolerate spontaneous breathing trials because of persistently elevated peak inspiratory pressures.

His hospital course was complicated by ventilator-associated pneumonia and persistent loculated pleural effusions. Initial blood cultures obtained on hospital day 11 demonstrated no growth. Subsequent respiratory cultures grew *Enterobacter cloacae* complex, while blood cultures obtained on hospital day 20 were positive for *Serratia marcescens*. Antimicrobial therapy included sequential treatment with cefepime and vancomycin, followed by piperacillin-tazobactam and meropenem. Clinical improvement was ultimately observed following completion of a one-week course of levofloxacin.

**Figure 4:**
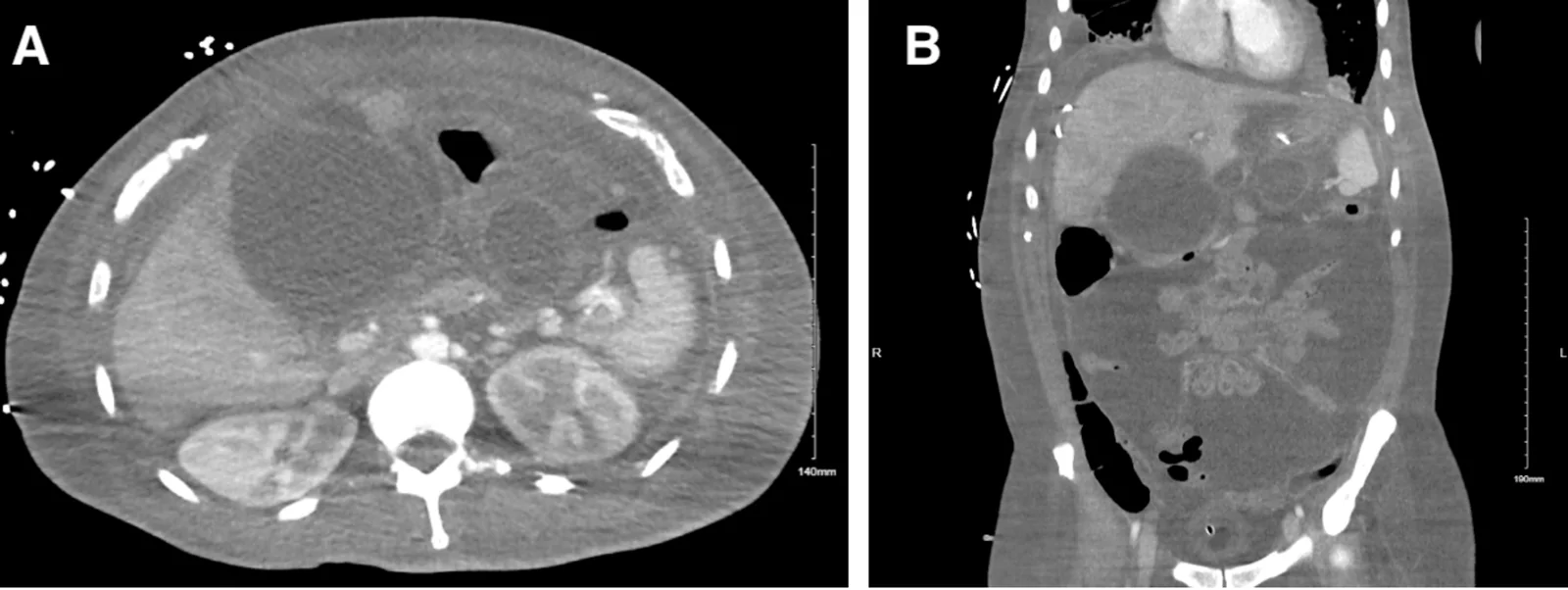
CT of the Abdomen and Pelvis Demonstrating Loculated Ascites and Peritoneal Drain Placement (A) Axial contrast-enhanced CT of the abdomen and pelvis demonstrating a large perihepatic loculated ascitic collection. (B) Coronal contrast-enhanced CT of the abdomen and pelvis showing multiple loculated intra-abdominal fluid collections, with a pigtail drainage catheter in the right upper quadrant demonstrating minimal output.

On hospital day 18, interventional radiology performed a left-sided thoracentesis and placed a pigtail drainage catheter within the right upper quadrant abdominal collection. Although fluid samples were successfully obtained, no solid tissue specimen could be collected. Cytologic analysis of the pleural and abdominal fluid collections was negative for malignant cells and therefore did not provide confirmatory tissue at the receiving institution despite the previously established diagnosis at the outside hospital. Repeat contrast-enhanced CT of the abdomen and pelvis obtained on hospital day 20 demonstrated the pigtail catheter within the right upper quadrant fluid collection and multiple perihepatic fluid collections without a suitable solid tissue target for biopsy (Figure 4).

EBUS performed on hospital day 23 failed to identify significant lymph node disease or suitable tissue targets for biopsy. Consultation with cardiothoracic surgery on hospital day 25 determined that pleural biopsy would carry substantial procedural risk with limited anticipated diagnostic benefit.

**Figure 5:**
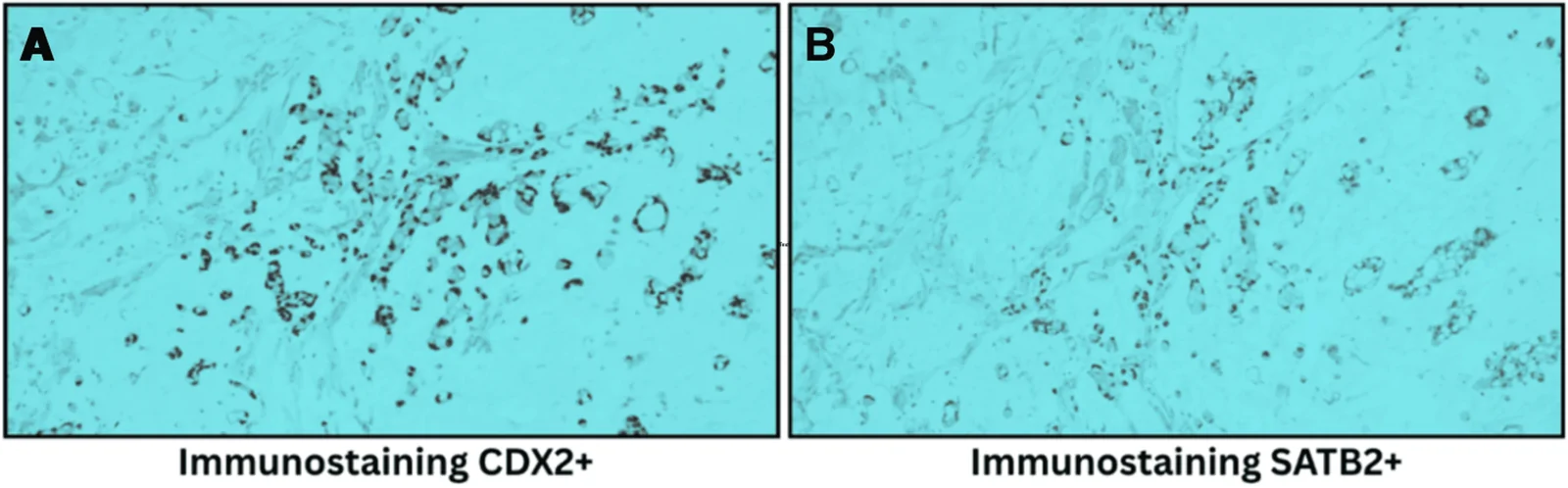
Immunohistochemical Staining of the Peritoneal Lesion (A) Positive CDX2 staining and (B) positive SATB2 staining in the peritoneal lesion. The expression of CDX2 and SATB2 supports lower gastrointestinal differentiation of the high-grade mucinous adenocarcinoma.

**Figure 6:**
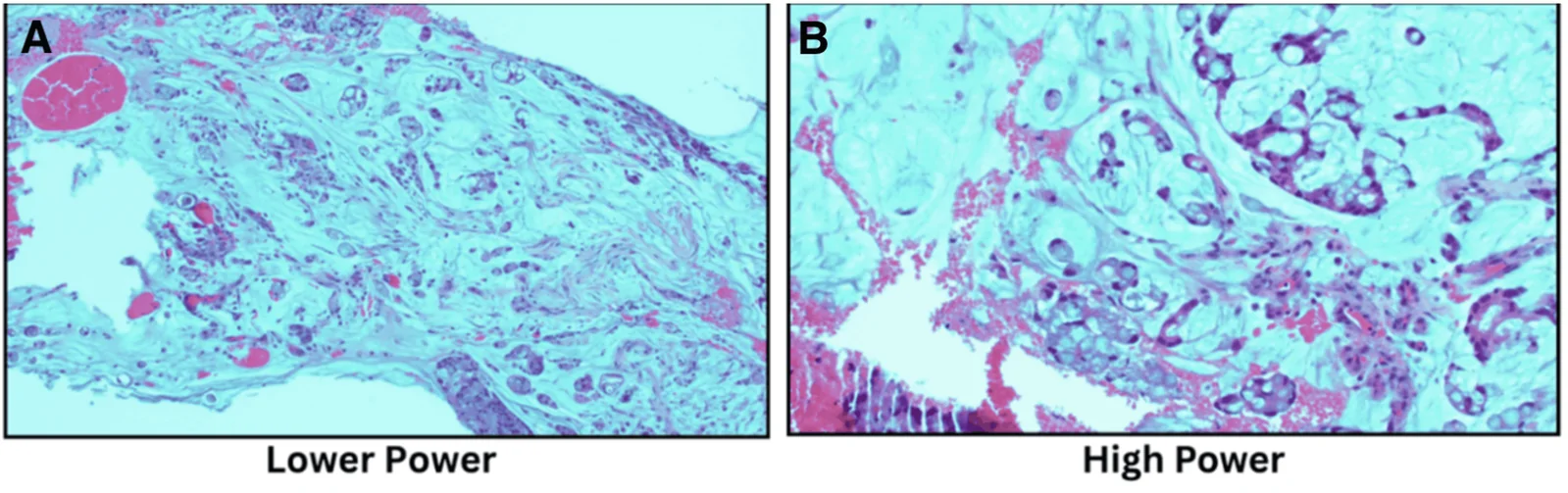
Histopathologic Features of Pseudomyxoma Peritonei (A) Low-power (10×) and (B) high-power (20×) microscopic views showing abundant extracellular mucin pools characteristic of high-grade mucinous adenocarcinoma of the peritoneum.

Because the complete outside pathology report and original histologic slides were unavailable for review following transfer, additional tissue sampling was pursued to confirm the diagnosis and further characterize the tumor. Thoracentesis, abdominal fluid drainage, and EBUS-guided sampling were nondiagnostic. Diagnostic laparoscopy was therefore performed on hospital day 30, demonstrating extensive peritoneal deposits and dense intra-abdominal adhesions, and the disease was considered surgically unresectable. Histopathologic evaluation demonstrated high-grade mucinous adenocarcinoma with signet-ring cells. Immunohistochemical staining demonstrated positivity for CK7, CK20, CDX2, and SATB2, supporting lower gastrointestinal differentiation (Figures 5-6). Esophagogastroduodenoscopy and colonoscopy performed in November 2024 did not identify a primary gastrointestinal malignancy. Although the immunophenotype supported lower gastrointestinal differentiation, a definitive primary tumor site could not be established based on the available clinical, operative, and pathologic findings. 

Following multidisciplinary discussion regarding prognosis and treatment options, the patient elected to proceed with palliative systemic chemotherapy. He received one cycle of FOLFOXIRI (5-fluorouracil, leucovorin, oxaliplatin, and irinotecan) from hospital days 37-39. Bevacizumab was withheld because of concern for bowel perforation in the setting of extensive intra-abdominal disease. Although an additional cycle was considered, no further chemotherapy was administered because the patient remained ventilator-dependent, developed *Enterobacterales* and *Serratia marcescens* bacteremia requiring vasopressor support, and continued to experience progressive clinical deterioration.

## Discussion

PMP is a rare clinicopathologic syndrome characterized by progressive accumulation and dissemination of mucinous ascites throughout the peritoneal cavity, most commonly originating from an appendiceal mucinous neoplasm [4]. The estimated incidence is approximately two to four cases per million individuals annually, making PMP an uncommon but clinically significant malignancy [5]. Classic symptoms include progressive abdominal distension, abdominal or pelvic pain, early satiety, and gastrointestinal dysfunction secondary to increasing intra-abdominal tumor burden [6]. In contrast to the typically indolent clinical course of PMP, the present case demonstrated rapid progression, radiographic findings concerning for thoracic involvement, and high-grade histopathology, highlighting the substantial heterogeneity that exists within this disease spectrum.

A particularly notable feature of this case was the patient's atypical presenting symptoms, which preceded recognition of the underlying malignancy. Gynecomastia, ejaculatory dysfunction, pelvic pain, and hydrocele formation are not recognized manifestations of PMP and initially directed evaluation toward endocrine and genitourinary etiologies rather than an intra-abdominal neoplasm. Although these symptoms preceded the diagnosis of PMP, a causal relationship cannot be established. Previous reports have described unusual presentations of PMP, including scrotal masses, inguinal hernias, uterine prolapse, and presumed ovarian pathology; however, the constellation of symptoms observed in this patient has not been widely reported [7-10]. The absence of classic abdominal manifestations likely contributed to the delayed diagnosis, which was ultimately established at exploratory laparotomy before transfer to the receiving institution. Similar diagnostic challenges have been described in other reports of PMP presenting with nonspecific or atypical symptoms, underscoring the importance of maintaining a broad differential diagnosis when persistent, unexplained symptoms fail to respond to conventional evaluation [10]. The pathogenesis of PMP remains incompletely understood. Although *Helicobacter pylori* is a well-established risk factor for certain gastric malignancies [11], microbial factors have also been investigated for their potential role in the biology of PMP, including possible effects on the tumor microenvironment and signaling pathways [12,13]. However, no causal relationship between *Helicobacter pylori* infection and PMP has been established, and the relevance of these findings to the present case remains uncertain.

Another important aspect of this case was the reported unremarkable abdominal MRI performed in January 2024. According to the outside radiology report, the MRI was interpreted as unremarkable; however, the original MRI images were unavailable for independent review. Repeat CT imaging obtained approximately three months later demonstrated substantial disease burden, including loculated ascites and a large abdominal mass. Although both MRI and CT are used in the evaluation of PMP, CT remains the preferred imaging modality because of its ability to identify mucinous deposits, omental caking, visceral scalloping, and the overall extent of peritoneal involvement [19]. Because the original MRI images were unavailable for independent review, it remains uncertain whether the discrepancy between the reported MRI findings and the subsequent CT reflects early disease, differences in imaging technique or protocol, or variability in radiologic interpretation. Nevertheless, this case underscores the importance of repeat imaging and further evaluation when symptoms progress despite an initially nondiagnostic imaging study. 

Histopathologic evaluation confirmed high-grade mucinous adenocarcinoma with signet-ring cells and positive CK7, CK20, CDX2, and SATB2 immunostaining (Figures 5-6). These findings support lower gastrointestinal differentiation but do not establish a specific primary site. Although an appendiceal origin is most commonly associated with PMP, esophagogastroduodenoscopy and colonoscopy performed in November 2024 did not identify a primary gastrointestinal malignancy, and a definitive primary tumor site could not be established based on the available clinical, operative, and pathologic information. Because the complete outside pathology report was unavailable for review, standardized PSOGI classification of the peritoneal disease could not be confirmed. Expression of CDX2 and SATB2 (Figure 5) strongly supports a gastrointestinal, particularly appendiceal or colorectal, origin and is frequently used to characterize mucinous neoplasms associated with PMP [17]. The presence of signet-ring cells is associated with more aggressive tumor biology and poorer prognosis, consistent with this patient's rapidly progressive clinical course. Although long-term survival has been reported following treatment of PMP, particularly among patients with low-grade disease and complete cytoreduction [14], high-grade disease is associated with increased recurrence rates, reduced responsiveness to therapy, and significantly poorer survival outcomes [15,16]. The present case illustrates the aggressive end of the PMP disease spectrum and underscores the prognostic significance of high-grade histology and signet-ring cell morphology.

Radiographic findings concerning for thoracic involvement represented another unusual feature of this case. PMP is generally confined to the peritoneal cavity, and pleural extension has been reported only rarely [18]. Several mechanisms have been proposed to explain thoracic dissemination, including hematogenous spread, lymphatic spread, and transdiaphragmatic migration of mucin-producing tumor cells [18]. Although the imaging findings were concerning for thoracic extension, histologic confirmation was not obtained. Pleural fluid cytology was repeatedly negative for malignant cells, EBUS failed to identify a suitable biopsy target, and pleural biopsy was considered prohibitively high risk. Alternative explanations for the pleural effusions, including severe pneumonia, systemic inflammation, and critical illness, cannot be excluded. Given the patient's extensive ascites and persistent pleural effusions, transdiaphragmatic or lymphatic dissemination appears most plausible. The radiographic thoracic abnormalities and associated pleural effusions substantially complicated management by impairing respiratory mechanics, limiting effective drainage, and contributing to prolonged ventilator dependence. The patient's respiratory status was further complicated by ventilator-associated pneumonia caused by *Enterobacter cloacae* complex and *Serratia marcescens*. Collectively, these findings suggest that radiographic thoracic abnormalities in patients with advanced PMP warrant careful evaluation, although the precise etiology of the pleural findings in this patient could not be definitively established.

Obtaining confirmatory tissue and complete histopathologic characterization at the receiving institution proved challenging. The initial diagnosis of PMP had already been established during the exploratory laparotomy performed at the outside institution before transfer, where ascitic-fluid cytology was positive for malignant cells, and an intraoperative biopsy demonstrated mucinous adenocarcinoma. However, the complete outside pathology report was unavailable for review after transfer. Subsequent minimally invasive procedures, including thoracentesis, abdominal fluid drainage, and EBUS-guided sampling, failed to provide adequate tissue for confirmatory histopathologic evaluation and immunophenotypic characterization. Pleural and abdominal fluid samples obtained at the receiving institution were repeatedly negative for malignant cells despite advanced disease. These later negative findings did not negate the previously established diagnosis and likely reflect the recognized sampling limitations of mucinous neoplasms, as mucinous collections are often paucicellular and may yield acellular mucin without representative neoplastic cells [20]. Diagnostic laparoscopy subsequently provided adequate tissue to confirm the previously established diagnosis and to permit comprehensive histopathologic and immunophenotypic characterization of the tumor. This case highlights the importance of obtaining adequate tissue when prior pathology is unavailable for review or when additional histopathologic and immunophenotypic characterization is necessary to guide treatment planning.

CRS-HIPEC remains the accepted standard of care for appropriately selected patients with PMP [21]. This approach has significantly improved long-term survival and disease control compared with historical debulking procedures [22]. In this case, diagnostic laparoscopy demonstrated extensive unresectable peritoneal disease. Additional factors limiting operative candidacy included ventilator-dependent hypoxemic and hypercapnic respiratory failure, declining functional status, severe chronic illness-related malnutrition, radiographic findings concerning for thoracic involvement, and poor overall clinical condition. Nutritional assessment documented greater than 20% estimated weight loss over one year, energy intake below 50% of estimated requirements for more than one month, severe orbital fat depletion, and severe temporal and clavicular muscle wasting. The patient required enteral nutrition via nasogastric tube, which at the time of assessment provided only approximately 36% of estimated caloric requirements and 28% of estimated protein requirements. Although a formal Eastern Cooperative Oncology Group (ECOG) performance status and Peritoneal Cancer Index (PCI) were not documented, these findings, together with unresectable disease and prolonged ventilator dependence, supported the multidisciplinary determination that CRS-HIPEC was not appropriate. Furthermore, evidence suggests that the survival benefit of CRS-HIPEC is reduced in patients with high-grade disease and extra-abdominal extension [15,22,23]. Current peritoneal surface malignancy guidelines generally regard extraperitoneal disease as a major limitation to curative surgical intervention [23]. Following multidisciplinary discussion, the patient received one cycle of palliative FOLFOXIRI; however, no further systemic therapy was administered because of persistent ventilator dependence, progressive clinical deterioration, and subsequent bacteremia requiring vasopressor support. Consequently, this case highlights the narrow therapeutic window that exists in aggressive PMP and emphasizes the importance of early recognition and referral to specialized centers before progression renders definitive treatment infeasible.

Overall, this case illustrates several uncommon features, including atypical endocrine and genitourinary presenting symptoms, initially nondiagnostic imaging, radiographic findings concerning for thoracic involvement, repeated nondiagnostic cytologic studies, and an unusually aggressive clinical course. These findings reinforce the importance of maintaining a broad differential diagnosis, obtaining adequate tissue for histopathologic confirmation when minimally invasive sampling is nondiagnostic, and facilitating early referral to specialized peritoneal surface malignancy centers before disease progression limits potentially curative treatment.

A chronological timeline of the patient's clinical course is presented in Table 1.

**Table 1:** Chronological Timeline of the Patient's Clinical Course Chronological summary of the patient's clinical presentation, diagnostic evaluation, disease progression, therapeutic interventions, and clinical outcome from initial symptom onset in 2023 through hospital day 55. The timeline highlights key imaging studies, operative findings, diagnostic procedures, and treatment milestones relevant to the diagnosis and management of high-grade pseudomyxoma peritonei. CRS-HIPEC, cytoreductive surgery combined with hyperthermic intraperitoneal chemotherapy; CT, computed tomography; CTA, computed tomography angiography; EBUS, endobronchial ultrasound; MRI, magnetic resonance imaging

Time Point	Event
2023	Initial presentation with progressive gynecomastia, ejaculatory dysfunction, and pelvic pain.
January 2024	MRI abdomen/pelvis reported as unremarkable.
April 2024	CT abdomen/pelvis demonstrated loculated ascites and a large abdominal mass.
June 2024	Progressive abdominal distension and unintentional weight loss.
November 2024	Esophagogastroduodenoscopy and colonoscopy performed; no primary gastrointestinal malignancy identified.
February 2025	Exploratory laparotomy at outside institution established diagnosis of pseudomyxoma peritonei with high-grade mucinous adenocarcinoma; approximately 11 L of malignant ascites evacuated.
Hospital day -3	CTA chest demonstrated bilateral pleural effusions, mediastinal abnormalities, and findings concerning for thoracic involvement.
Hospital day 1	Hospitalized with acute hypercapnic respiratory failure requiring endotracheal intubation.
Hospital day 11	Transferred to Arrowhead Regional Medical Center for evaluation for CRS-HIPEC.
Hospital days 18-23	Thoracentesis, abdominal drainage, and EBUS performed; cytology and tissue sampling remained nondiagnostic.
Hospital day 30	Diagnostic laparoscopy confirmed unresectable high-grade mucinous adenocarcinoma with signet-ring cells.
Hospital days 37-39	One cycle of palliative FOLFOXIRI (5-fluorouracil, leucovorin, oxaliplatin, and irinotecan) administered.
Hospital days 40-54	No further chemotherapy administered because of persistent ventilator dependence, *Enterobacterales* and *Serratia marcescens* bacteremia requiring vasopressor support, and progressive clinical deterioration.
Hospital day 55	Patient died despite multidisciplinary management.

## Conclusions

PMP is a rare malignancy that may present with atypical symptoms and initially inconclusive imaging findings, contributing to delayed diagnosis. This case highlights the limitations of cytology and minimally invasive tissue sampling in mucinous peritoneal disease. Although the diagnosis was initially established by exploratory laparotomy at an outside institution, repeated minimally invasive procedures after transfer failed to provide confirmatory tissue until diagnostic laparoscopy demonstrated extensive unresectable disease and high-grade mucinous adenocarcinoma with signet-ring cells. Adequate tissue sampling may therefore be required to confirm the diagnosis, establish histologic grade, perform immunohistochemical characterization, and guide treatment planning.

The patient's unusually aggressive clinical course, characterized by severe cachexia, ventilator-dependent respiratory failure, declining functional status, and radiographic findings concerning for thoracic involvement, ultimately precluded treatment with cytoreductive surgery and hyperthermic intraperitoneal chemotherapy (CRS-HIPEC) and limited systemic therapy to a single cycle of palliative FOLFOXIRI. Early recognition and timely referral to specialized peritoneal surface malignancy centers are essential, as progression to advanced disease and deterioration in functional status may preclude potentially curative treatment. This case underscores the importance of maintaining a high index of suspicion for PMP in patients with unexplained progressive ascites or persistent atypical abdominal symptoms and highlights the value of multidisciplinary evaluation in optimizing diagnostic accuracy and treatment planning.
